# Single and double stereoselective fluorination of (*E*)-allylsilanes

**DOI:** 10.1186/1860-5397-3-34

**Published:** 2007-10-25

**Authors:** Marcin Sawicki, Angela Kwok, Matthew Tredwell, Véronique Gouverneur

**Affiliations:** 1 University of Oxford, Chemistry Research Laboratory, Mansfield Road, Oxford, OX1 3TA, UK

## Abstract

Acyclic allylic monofluorides were prepared by electrophilic fluorination of branched (*E*)-allylsilanes with Selectfluor. These reactions proceeded with efficient transfer of chirality from the silylated to the fluorinated stereocentre. Upon double fluorination, an unsymmetrical ethyl *syn*-2,5-difluoroalk-3-enoic ester was prepared, the silyl group acting as an *anti* stereodirecting group for the two C-F bond forming events.

## Findings

**Scheme 1:**
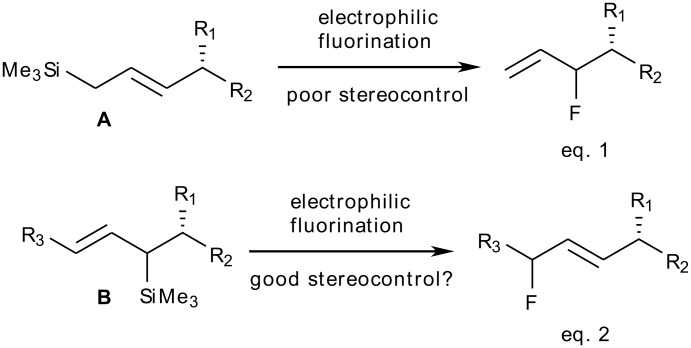
Fluorination of branched allylsilanes **A** and **B**

Asymmetric C-F bond formation continues to challenge chemists, inspiring the development of increasingly effective protocols for stereocontrolled fluorination. [1–11] Studies from our laboratory illustrated that allylsilanes undergo electrophilic fluorination to afford allylic fluorides with clean transposition of the double bond. Using chiral cyclic allylsilanes, these experiments have culminated in efficient methods for the asymmetric synthesis of monofluorinated cyclitols or vitamin D3 analogues. [12–15] The key step of these syntheses is a highly efficient diastereoselective fluorodesilylation. We encountered more difficulties with the fluorination of acyclic allylsilanes **A** constructed by metathetic coupling of allyltrimethysilane with chiral olefinic partners (eq. 1, Scheme 1). Although high yielding, the electrophilic fluorination of these substrates suffered from a poor level of diastereocontrol, thereby limiting the synthetic value of these reactions.[16–17] The absence of a silylated stereogenic centre is likely to be responsible for the poor stereocontrol observed upon fluorination of these substrates. We envisaged that the fluorination of (*E*)-allylsilanes **B**, featuring a silylated stereogenic centre, might be a superior transformation to control the configuration of the emerging fluorine-bearing centre (eq. 2, Scheme 1). This working hypothesis is supported by the well-accepted model, which accounts for the observed transfer of chirality when reacting allylsilanes **B** with electrophiles other than fluorine. [18–21] Chiral allylsilanes **B** are known to act as useful carbon nucleophile equivalents in highly stereoselective condensation reactions with a large range of electrophiles leading to the construction of C-C, C-O, C-N or C-S bonds. [22–27] With the nitrogen-based electrophile NO_2_BF_4_, this methodology delivers acyclic (*E*)-olefin dipeptide isosteres featuring two allylic stereocentres.[28–29]

Herein, we report our investigation into the fluorination of (*E*)-allylsilanes of general structure **B**. A highly efficient and stereoselective synthesis of alkenes featuring bis-allylic stereocentres, one of them being fluorinated, emerged from this study. Significantly, alkenes flanked by two allylic fluorinated stereogenic centres are also accessible upon double electrophilic fluorination of (*E*)-allylsilanes substituted with an ester group.

The synthesis of the allylsilanes (±)-**1a-i** featuring an ester or alcohol group was carried out according to the procedure reported by Panek and co-workers.[30] See Supporting Information File 1 for full experimental data. The fluorinations were carried out at room temperature in CH_3_CN in the presence of 1.0 eq. of NaHCO_3_ and 1.5 eq. of Selectfluor [1-chloromethyl-4-fluoro-1,2-diazoniabicyclo[2.2.2]octane bis(tetrafluoroborate)]. The reactivity of the (*E*)-allylsilanes **1a-d** possessing a single stereogenic centre was surveyed in priority to probe how structural variations on these substrates influence the *E/Z* selectivity of the resulting allylic fluorides (Table 1). For the (*E*)-allylsilane **1a**, the allylic fluoride **2a** was obtained in 81% yield as a roughly 1/1 mixture of *E*/*Z* geometrical isomers (entry 1). The structurally related (*E*)-allylsilane **1b** possessing the primary alcohol group underwent fluorination with a lower yield of 64%, delivering preferentially the *E*-isomer with poor selectivity (entry 2). The fluorination of allylsilanes featuring the primary alcohol gave, in addition to the desired product, various amounts of O-trimethylsilylated 5-fluoroalk-3-enols. The presence of the *gem*-dimethyl group on the starting silanes **1c** and **1d** drastically improved the stereochemical outcome of the fluorination. Compounds *E*-**2c** and *E*-**2d** were formed in 95% and 70% yield respectively, with no trace of *Z*-isomer detectable in the crude reaction mixture (entries 3 and 4).

**Table 1:** Fluorodesilylation of (*E*)-allylsilanes (±)-**1a-d^a^**

Entry	(*E*)-Allylsilane	Major product	Yield	*E:Z* ^b^

1	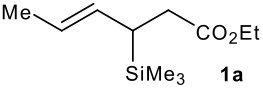	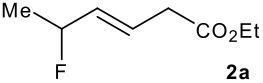	81%	1.3:1
2	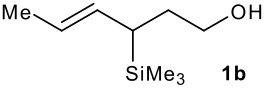	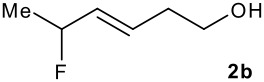	64%	3:1
3	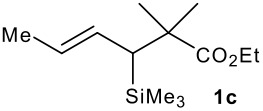	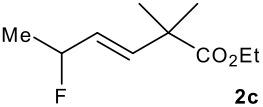	95%	>20:1
4	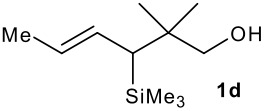	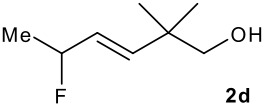	70%	>20:1

a: 1eq. NaHCO_3_, 1.5 eq. Selectfluor, CH_3_CN, rt; b: ratio determined by ^19^F NMR on crude reaction mixtures

**Scheme 2:**
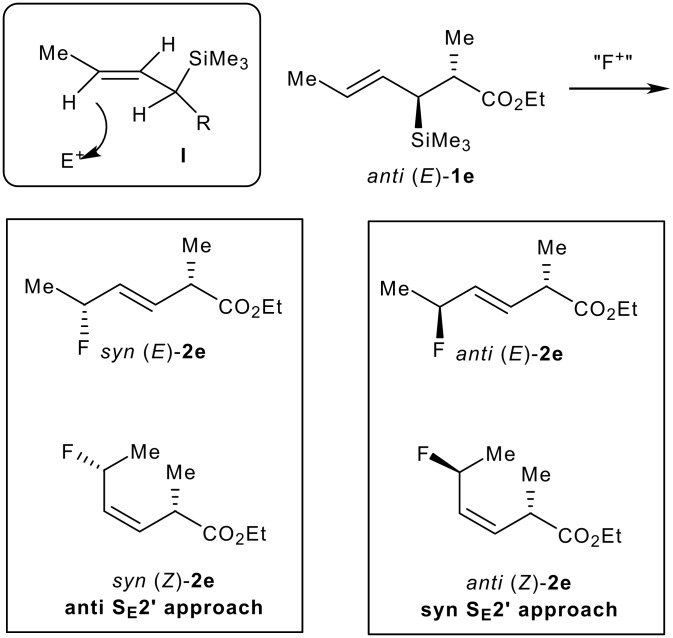
Fluorination of *anti* (*E*)-**1e**

When a second stereogenic centre is present on the starting (*E*)-allylsilane, up to four stereoisomers may be formed upon fluorination. This is illustrated in Scheme 1 with the fluorodesilylation of *anti* (*E*)-**1e**. For substitution reactions (S_E_2') of allylsilanes such as *anti* (*E*)-**1e**, with electrophiles other than "F^+^", an *anti* approach with respect to the silyl group prevails with preferential formation of the *syn* (*E*) isomer. [18–21] This stereochemical outcome suggests that the major reaction pathway involves the reactive conformer I leading, after addition of the electrophile, to a carbocationic intermediate which undergoes rapid elimination prior to bond rotation (Scheme 2).

Subsequent experiments focused on the fluorination of *anti* and *syn* (*E*)-allysilanes **1e-i** to study the effect of silane configuration on diastereoselection (Table 2). Upon fluorination of *anti*-**1e**, the allylic fluoride **2e** was formed in 95% yield as a diastereomeric mixture of both *syn*-**2e** and *anti*-**2e** isomers. The high d.r. [19:1] suggested that the transfer of chirality (*anti* approach of Selectfluor) from the silylated to the fluorinated stereocentre was very efficient. A third allylic fluoride was detected in the crude mixture and its structure was tentatively assigned as *syn* (*Z*)-**2e** (entry 1). The benzyl-substituted allylsilane *anti*-**1f** was fluorinated in 90% yield with a similar sense and level of stereocontrol (entry 2). Excellent transfer of chirality was also observed for the fluorination of *anti* (*E*)-**1g** featuring the primary alcohol group but the yield was significantly lower (entry 3). *Syn*-**1h**, which was used contaminated with *anti*-**1h** [d.r. = 9:1], was fluorinated with an overall yield of 96% delivering a mixture of four stereoisomeric allylic fluorides (entry 4). For this reaction, erosion of stereointegrity resulting from alternative reacting conformation, *syn* approach of the fluorinating reagent with respect to the silyl group, or bond rotation prior to elimination, was detectable but minimal. A similar stereochemical trend was observed for the alcohol *syn* (*E*)-**1i** (entry 5). The stereochemical assignment of compounds **2a-i** was assigned by analogy with the nitration of identical (*E*)-allylsilanes as reported by Panek. [22–27]

**Table 2:** Fluorodesilylation (*E*)-allylsilanes **1e-i^a^**

Entry	(*E*)-Allylsilane *anti:syn*	Major product	Yield	Syn:anti^b^ *E:Z*(syn)^b^ *E:Z*(anti)^b^

1	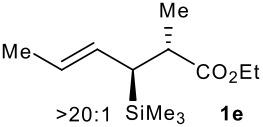	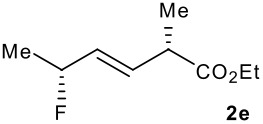	95%	19:1 15:1 >20:1
2	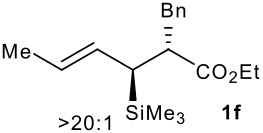	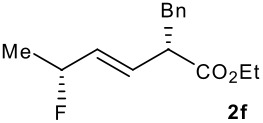	90%	>20:1 11:1 >20:1
3	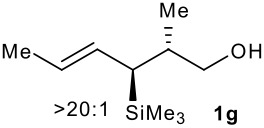	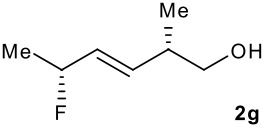	66%	>20:1 15:1 >20:1
4	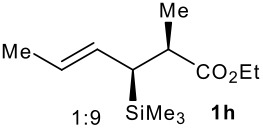	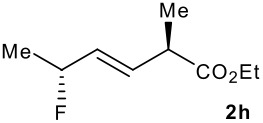	96%	1:6 >20:1 11:1
5	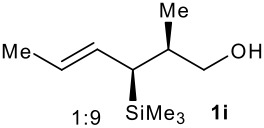	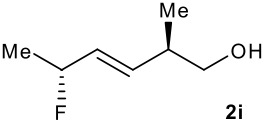	86%	1:8 14:1 10:1

a: 1eq. NaHCO_3_, 1.5 eq. Selectfluor, CH_3_CN, rt; b: ratio determined by ^19^F NMR on crude reaction mixtures

This chemistry offers the unique opportunity to access alkenes flanked with two allylic and stereogenic fluorinated centres upon double electrophilic fluorination of (*E*)-allylsilanes featuring an ester group. Although undoubtedly versatile for further functional manipulation, this structural motif is extremely rare with only two symmetrical variants reported in the literature. [31–32] The prospect of validating a more general strategy for the preparation of both symmetrical and unsymmetrical alkenes doubly flanked by fluorinated allylic stereocentres prompted us to challenge our methodology with the preparation of the unsymmetrical difluorinated alkenoic ester **3**. This compound was subsequently converted into a known symmetrical difluorinated alkene for which the relative stereochemistry was unambiguously identified by X-ray analysis.[31] This line of conjecture allowed us to verify our stereochemical assignments.

**Scheme 3:**
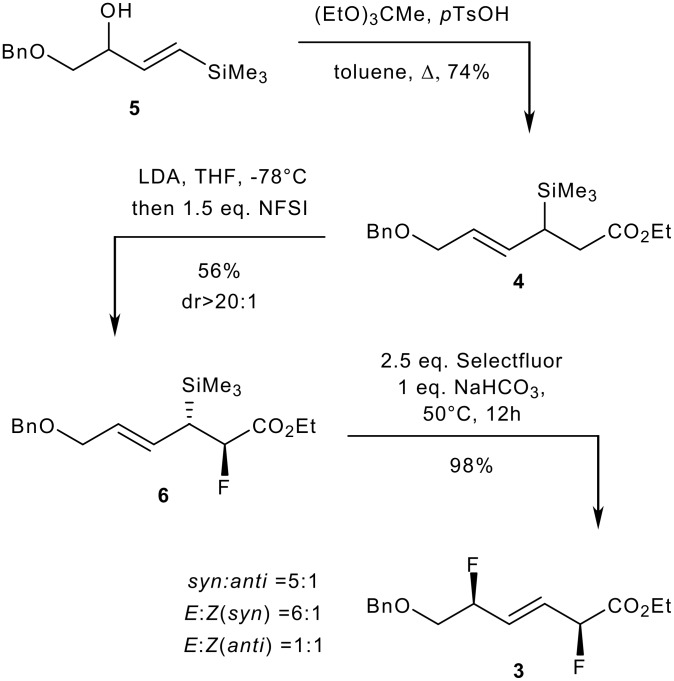
Double electrophilic fluorination of (*E*)-**4**

We investigated the feasibility of the double fluorination with (*E*)-allylsilane **4** prepared from (*E*)-vinylsilane **5** [33] via a [3,3] sigmatropic rearrangement. As anticipated and much to our delight, the doubly fluorinated alkene **3** was obtained through a succession of two electrophilic fluorinations. The electrophilic α-fluorination of the ester **4** was performed by treatment with LDA at -78°C followed by addition of *N*-fluorobenzenesulfonimide [34] (NFSI). The d.r. for this first fluorination was excellent (>20:1). The subsequent electrophilic fluorodesilylation of the resulting fluorinated silane **6** delivered **3** in excellent yield with no trace of side-products. In comparison with allylsilanes **1a-i**, the fluorodesilylation of **6** was more demanding and required higher temperature to reach completion. Under these conditions, the level of stereocontrol of the second fluorination was moderate (Scheme 3).

**Scheme 4:**
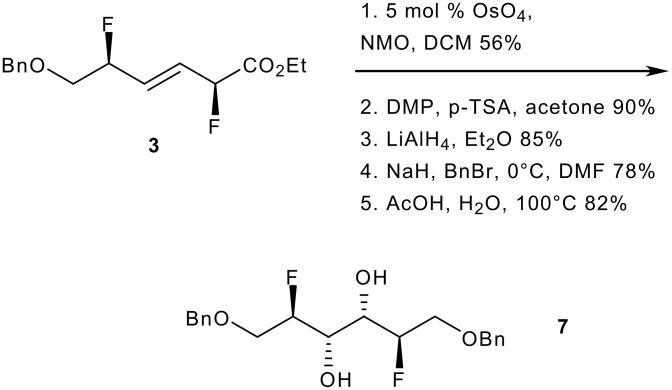
Conversion of **3** into diol **7**

To unambiguously confirm the stereochemistry of *syn* (*E*)-**3** [major diastereomer], this compound was converted into the known symmetrical difluorinated alkene **7** (Scheme 4). The key steps necessary to perform this conversion were a dihydroxylation, the reduction of the ester group and the benzylation of the resulting primary alcohol. Preliminary work revealed that the order of steps was important and that protecting group manipulations were required for clean product outcome. The *cis*-dihydroxylation of **3** was performed employing NMO and catalytic OsO_4_ in DCM.[35] In the event, the diastereoselectivity was controlled by the two fluorine substituents. Four successful operations separated the newly formed unsymmetrical diol from **7**, namely the protection of the diol as an acetonide, the reduction of the ester, the benzylation of the resulting primary alcohol and a final deprotection step. The spectroscopic data of compound **7** were identical to the ones of a sample prepared independently according to the procedure reported by O'Hagan.[31] This observation establishes the relative configuration as drawn in Scheme 2 and Scheme 3, and supports our hypothesis that the sense of stereocontrol for the fluorinations of **1e-i** is in line with related nitrations reported by Panek.[28]

In conclusion, the stereoselective fluorination of (*E*)-allylsilanes featuring a silylated stereogenic centre was found to be a useful reaction for the preparation of allylic fluorides, the silyl group acting as an efficient stereodirecting group. Notably, this methodology enables the preparation of unsymmetrical alkenes doubly flanked with fluorinated stereogenic centres. This result is significant as only symmetrical derivatives are accessible with the method reported to date.[31–32]

## Supporting Information

File 1 Single and double stereoselective fluorination of (*E*)-allylsilanes. Full experimental data and procedures
