# Supplementary material for: Single and double stereoselective fluorination of (E)-allylsilanes
Source: Beilstein J Org Chem. 2007 Oct 25;3:34. doi: 10.1186/1860-5397-3-34 (PMC2151074; doi:10.1186/1860-5397-3-34)
Supplement: File 1 — Single and double stereoselective fluorination of (E)-allylsilanes. Full experimental data and procedures [file Beilstein_J_Org_Chem-03-34-s001.doc]

**Single and Double Stereoselective Fluorination of (*E*)-Allylsilanes**

Marcin Sawicki, Angela Kwok, Matthew Tredwell, Véronique Gouverneur^*^

*University of Oxford, Chemistry Research Laboratory, Mansfield Road, Oxford, UK, OX1 3TA.*

**Additional Material**

**General Information**

^1^H NMR spectra were recorded in deuterated solvents by using Bruker AV400 or AV500 spectrometers, calibrated using residual protonated solvent as an internal reference. ^13^C NMR spectra were recorded in deutrated solvent by using Bruker AV400 or AV500 spectrometers. ^19^F NMR spectra were recorded on a Bruker AV400 spectrometer. Chemical shifts (δ) are reported in parts per million (ppm) and coupling constants (*J*) are measured in hertz (Hz). The following abbreviations are used to describe multiplicities s = singlet, d = doublet, t = triplet, q = quartet, br = broad, m = multiplet. NMR were processed in either MestRe-C or ACD/SpecManager. IR spectra were recorded on a Bruker Tensor 27 FTIR spectrometer. Absorption bands are measured in wavenumbers (cm^-1^) and only peaks on interest are reported. Mass spectra (*m/z*) and HRMS were recorded on a Micromass LCT in ElectronSpray Ionisation (ESI), Field Ionization (FI) mode or on a Micromass AutoSpec (CI^+^). All reactions requiring anhydrous conditions were conducted in dried apparatus under an inert atmosphere of argon or nitrogen. Solvents were dried and purified before use, according to standard procedures. Thin layer chromatography (TLC) was performed using Merck Kieselgel 60F_254_ and visualized with a UV lamp or by staining with KMnO_4_. Column chromatography was carried out over Merck silica gel C60 (40-60 µm) and eluents as indicated.

**Compounds 1a, 1e and 1h were synthesized according to reported procedures.^1^**

### (*E*)-3-(Trimethylsilyl)hex-4-en-1-ol (1b).

A solution of (*E*)-3-(trimethylsilyl)hex-4-enoate (897mg, 4.2mmol) in Et_2_O (21ml) was added dropwise to a slurry of LiAlH_4_ (800mg, 21mmol) in Et_2_O (21ml) and allowed to stir at room temperature for 4hrs. The reaction was quenched by the addition of a saturated solution of NH_4_Cl at 0°C. The aqueous phase was extracted with Et_2_O. The combined organic phases were dried over MgSO_4_ and the solvent removed *in vacuo*. Purification by silica gel chromatography (pentane: Et_2_O, 80:20; R_f_ = 0.19) afforded *(E)-*3-(trimethylsilyl)hex-4-en-1-ol (**1b**) (791mg, 84% yield). **^1^H NMR** (400 MHz, C_6_D_6_): δ -0.46 (s, 9H), 0.77 (br, 1H), 1.31-1.41 (m, 1H), 1.44-1.52 (m, 1H), 1.52-1.58 (m, 1H), 1.56 (d, *J*=5.7 Hz, 3H), 3.43 (ddd, *J*=10.4 Hz, 7.6 Hz, 6.4 Hz, 1H), 3.53 (ddd, *J*=10.4 Hz, 7.1 Hz, 4.6 Hz, 1H), 5.28 (dd, *J*=15.1 Hz, 7.4 Hz, 1H), 5.30 (dq, *J*=15.1 Hz, 5.7 Hz 1H); **^13^C NMR** (100 MHz, C_6_D_6_) δ -3.2 (3 x CH_3_), 18.2 (CH_3_), 29.7 (CH_2_), 32.2 (CH), 62.5 (CH_2_), 123.2 (CH), 132.3 (CH); **IR** (neat): 3333, 2956, 1248, 1034, 968, 837; **MS**: **HRMS**: calculated for C_9_H_20_NaOSi: m/z 195.1181, found: m/z 195.1186.

**(*E*)-2,2-Dimethyl-3-(trimethylsilyl)hex-4-enoate (1c).**

To a solution of diisopropylamine (1.57ml, 11.8mmol, 1.3 eq) in THF (9ml) at 0°C was added *n*-BuLi (2.5M, 4.4ml, 11.0mmol, 1.2eq) and allowed to stir for 30 min. The solution was cooled to -78°C and a solution of ethyl-(*E*)-2-methyl-3-trimethylsilylhex-4-enoate (2.052g, 9.0mmol) in THF (4.5ml) was added via a syringe. The temperature was gradually raised to -40°C over a period of 2hrs. After which time iodomethane (0.735ml, 11.8mmol, 1.3 eq) was added and the reaction mixture was allowed to warm to room temperature overnight. The reaction was quenched with saturated NH_4_Cl at 0°C. The aqueous phase was extracted with Et_2_O, the combined organic extracts were washed with saturated brine and dried over MgSO_4_, filtered and the solvent removed *in vacuo* to afford crude ethyl (*E*)-2-methyl-3-(trimethylsilyl)hex-4-enoate (**1c**) as a yellow oil. Purification by silica gel chromatography (40°-60° petroleum ether: Et_2_O, 9:1, R_f_ = 0.40) afforded (**1c**) 1.560g, 71% yield. **^1^H NMR** (400 MHz, CDCl_3_): δ -0.23 (s, 9H), 1.13 (s, 3H), 1.19 (s, 3H), 1.24 (t, *J*=7.1 Hz, 3H), 1.67 (d, *J*=4.9 Hz, 3H), 1.94 (d, *J*=10.2 Hz, 1H), 4.04 (dq, *J*=10.5 Hz, 7.1 Hz, 1H), 4.10 (dq, *J*=10.5 Hz, 7.1 Hz, 1H), 5.28 (dd, *J*=14.9 Hz, *J*=9.8 Hz, 1H), 5.30 (dq, *J*=14.9 Hz, 4.9 Hz, 1H); **^13^C NMR** (100 MHz, CDCl_3_): δ -0.9 (3 x CH_3_), 14.2 (CH_3_), 18.1 (CH_3_), 23.8 (CH_3_), 26.4 (CH_3_), 42.3 (CH), 44.2 (C), 60.2 (CH_2_), 126.1 (CH), 128.3 (CH), 178.3 (C); **IR** (neat): 2966, 1729, 1248, 1147, 971, 839; **HRMS**: calculated for C_13_H_26_O_2_Si: m/z 242.1702, found: m/z 242.1709.

**(*E*)-2,2-Dimethyl-3-(trimethylsilyl)hex-4-en-1-ol (1d).**

A solution of ethyl-(*E*)-2,2-dimethyl-3-(trimethylsilyl)hex-4-enoate (**1c**) (1.150g, 4.5mmol) in Et_2_O (22.5ml) was added to a slurry of LiAlH_4_ (860mg, 22.5mmol) in Et_2_O (22.5ml) dropwise, the reaction was allowed to stir at room temperature for 4hrs. The reaction was quenched by the addition of a saturated solution of NH_4_Cl at 0°C. The aqueous phase was extracted with Et_2_O, the combined organic extracts were washed with saturated brine and dried over MgSO_4_, filtered and the solvent removed *in vacuo*. Purification by silica gel chromatography (hexane: Et_2_O, 80:20; R_f_ = 0.35) afforded (**1d**) 772mg, 80% yield. **^1^H NMR** (400 MHz, C_6_D_6_): δ 0.06 (s, 9H), 0.86 (s, 3H), 0.96 (s, 3H), 1.20 (br, 1H), 1.56 (d, *J*=11.8 Hz, 1H), 1.57 (dd, *J*=6.2 Hz, 1.4 Hz, 3H), 3.12 (d, *J*=10.6 Hz, 1H), 3.26 (d, *J*=10.6 Hz, 1H), 5.15 (dq, *J*=14.9 Hz, 6.2 Hz, 1H), 5.45 (ddq, *J*=14.9 Hz, 11.8 Hz, 1.4 Hz, 1H; H_4_); **^13^C NMR** (100 MHz, C_6_D_6_): δ 0.4 (3 x CH_3_), 18.1 (CH_3_), 23.7 (CH_3_), 25.7 (CH_3_), 38.2 (C), 41.9 (CH), 72.2 (CH_2_), 125.0 (CH), 130.4 (CH); **IR** (neat): 3385, 1248, 1038, 973, 836. **HRMS**: calculated for C_11_H_24_OSi: m/z 223.1489, found: m/z 223.1493.

***anti*-(*E*)-Ethyl-2-methyl-3-(trimethylsilyl)hex-4-enoate (1f).**

To a solution of diisopropylamine (1.10ml, 7.8mmol, 1.3 eq) in THF (5.0ml) at 0°C was added *n*-BuLi (2.5M, 2.9ml, 7.2mmol, 1.2eq.), the reaction was allowed to stir for 30 min. The solution was cooled to -78°C and a solution of ethyl-(*E*)-3-trimethylsilylhex-4-enoate (1.28g, 6.0mmol) in THF (5ml) was added via a syringe. The light yellow solution was stirred for 30 min, followed by the dropwise addition of a solution of benzyl bromide (0.890ml, 7.5mmol, 1.2 eq) in THF (1 mL). The reaction mixture was allowed to stir and warm to room temperature overnight. The reaction was quenched by the addition of a saturated solution of NH_4_Cl at 0°C. The aqueous phase was extracted with Et_2_O. The combined organic phases were washed with saturated brine, dried over MgSO_4_, filtered and the solvent removed *in vacuo*. Purification by silica gel chromatography (hexane: Et_2_O, 9:1; R_f_ = 0.50) afforded (**1f**) as a colourless oil 1.480g, 81% yield, d.r >20:1. **^1^H NMR** (400 MHz, acetone-*d_6_*) δ -0.03 (s, 9H), 1.01 (t, *J*=7.1 Hz, 3H), 1.72 (d, *J*=4.8 Hz, 3H), 1.89-1.95 (m, 1H), 2.70 (t, *J*=11.6 Hz, 1H), 2.76 (ddd, *J*=11.6 Hz, 8.8 Hz, 3.0 Hz, 1H), 2.99 (dd, *J*=12.4 Hz, *J*=2.3 Hz, 1H), 3.90 (qd, *J*=7.1 Hz, 1.8 Hz*,* 1H), 5.36-5.48 (m, 3H), 7.14-7.19 (m, 2H), 7.22-7.28 (m, 2H); **^13^C NMR** (100 MHz, acetone-*d_6_*) δ -2.5 (3 x CH_3_), 13.9 (CH_3_), 17.8 (CH_3_), 37.0 (CH), 38.9 (CH_2_), 48.9 (CH), 59.8 (CH_2_), 125.4 (CH),126.4 (CH), 128.5 (2 x CH), 129.3 (2 x CH), 130.0 (CH), 140.5 (C), 174.6 (C); **IR** (neat): 2957, 1732, 1453, 1249, 1153, 839, 699; **HRMS**: calculated for C_18_H_29_O_2_Si: m/z 305.1931, found: m/z 305.1921.

**(*E)*-2-Methyl-3-(trimethylsilyl)hex-4-en-1-ol (1g).**

A solution of *anti*-ethyl-(*E)*-2-methyl-3-(trimethylsilyl)hex-4-enoate (1.030g, 4.5mmol) in Et_2_O (22.5ml) was added dropwise to a slurry of LiAlH_4_ (860mg, 22.5mmol) in Et_2_O (22.5ml) and allowed to stir at room temperature for 4hrs. The reaction was quenched by the addition of a saturated solution of NH_4_Cl at 0°C. The aqueous phase was extracted with Et_2_O. The combined organic phases were dried over MgSO_4_, filtered and the solvent removed *in vacuo*. Purification by silica gel chromatography (hexane: EtOAc, 80:20; R_f_ = 0.34) gave (**1g**) 757mg, 90% yield, dr >20:1. **^1^H NMR** (400 MHz, CDCl_3_): δ –0.35 (s, 9H), 0.89 (d, *J*=7.0 Hz, 3H) 1.50 (br, 1H), 1.64 (d, *J*=4.8 Hz, 3H), 1.69 (dd, *J*=9.9 Hz, 3.4 Hz, 1H), 1.84 (qtd,  *J*=7.0 Hz, 7.0 Hz, 3.4 Hz, 1H), 3.37 (dd,  *J*=10.6 Hz, 7.0 Hz, 1H), 3.41 (dd, *J*=10.6 Hz, 7.0 Hz, 1H), 5.22-5.38 (m, 2H); **^13^C NMR** (100 MHz, CDCl_3_): δ -2.1 (3 x CH_3_), 13.9 (CH_3_), 18.2 (CH_3_), 35.0 (CH), 35.5 (CH), 68.3 (CH_2_), 125.0 (CH), 127.9 (CH); **IR** (neat): 3334, 2958, 1248, 1033, 971, 836. **HRMS**: calculated for C_10_H_22_NaOSi: m/z 209.1332, found: m/z 209.1338.

***(E)*-2-Methyl-3-(trimethylsilyl)hex-4-en-1-ol (1i).**

A solution of *syn-*ethyl*-(E)*-2-methyl-3-(trimethylsilyl)hex-4-enoate (41 mg, 0.2 mmol) in Et_2_O (1.0 ml) was added dropwise to a slurry of LiAlH_4_ (35 mg, 1.0 mmol) in Et_2_O (1.0 ml) and allowed to stir at room temperature for 4hrs. The reaction was quenched by the addition of a saturated solution of NH_4_Cl at 0°C. The aqueous phase was extracted with Et_2_O. The combined organic phases were dried over MgSO_4_, filtered and the solvent removed *in vacuo*. Purification by silica gel chromatography (hexane: Et_2_O, 1:1; R_f_ = 0.4) afforded (**1i**) 31 mg, 84% yield, d.r = 9:1. **^1^H NMR** (400 MHz, acetone-d_6_): δ 0.02 (s, 9H), 0.98 (d, *J*=6.6 Hz, 3H), 1.48 (dd, *J*=9.9 Hz, 6.6 Hz, 1H), 1.65 (d, *J*= 4.8 Hz, 3H), 1.77-1.87 (m, 1H), 3.26 (ddd,  *J*=10.4 Hz, 7.8 Hz,5.6 Hz, 1H), 3.42 (t, *J*=5.3 Hz, 1H), 3.61 (dt, 10.4 Hz, 4.8 Hz, 1H), 5.26 (dq, *J*=15.2 Hz, 5.6 Hz, 1H), 5.33 (dd, *J*=15.2 Hz, 5.6 Hz, 1H); **^13^C NMR** (100 MHz, acetone-d_6_): δ -2.1 (3 x CH_3_), 13.9 (CH_3_), 18.2 (CH_3_), 35.0 (CH), 35.5 (CH), 68.3 (CH_2_), 125.0 (CH), 127.9 (CH); **IR** (neat): 3333, 2957, 1453, 1248, 1030, 97, 835. **HRMS**: calculated for C_10_H_22_NaOSi: m/z 209.1332, found: m/z 209.1336.

**General Procedure A for the electrophilic fluorodesilylation of esters.**

To a solution of substrate in acetonitrile (0.1 M) was added Selectfluor™ (1.5 eq.) and NaHCO_3_ (1eq.). The reaction mixture was allowed to stir at room temperature for 12hrs. The reaction was diluted with Et_2_O, filtered and the organic phase was washed with water. The organic phase was dried over MgSO_4_, filtered and the solvent removed *in vacuo*. (Caution: The corresponding allylic fluoride products are volatile, unstable on silica, alumina (basic and neutral) and decompose upon heating).

### Ethyl 5-fluorohex-3-enoate (2a).

General procedure A. 515mg, 81 % yield, *E/Z* = 1.3:1. **The *trans* isomer:** **^1^H NMR** (400 MHz, C_6_D_6_): δ 0.92 (t, *J=*7.1Hz, 3H), 1.11 (ddd, *J*=23.1 Hz, 6.4 Hz, 2.2 Hz, 3H), 2.76 (m,1H), 3.88 (q, *J*=7.1 Hz, 2H), 4.74 (dqd, *J*=48.2 Hz, *J*=6.4 Hz, *J*=6.4 Hz, 1H), 5.32-5.42 (m, 1H), 5.75 (dtd, *J*=15.6 Hz, 7.2 Hz, 4.1Hz, 1H); **^13^C NMR** (100 MHz, C_6_D_6_): δ 14.1 (CH_3_), 21.1 (d, *J*=24.4 Hz; CH_3_), 30.1 (CH_2_), 60.5 (CH_2_), 89.2 (d, *J*=163.8 Hz; CH), 125.2 (d , *J*=11.7 Hz; CH), 133.6 (d , *J*=19.6Hz; CH), 170.4 (C); **^19^F NMR** (376.5 MHz, C_6_D_6_) δ -166.6 (dqdd, *J*=48.2 Hz, 23.1Hz, 11.8Hz, 4.1Hz), **HRMS**: calculated for C_8_H_13_FNaO_2_ m/z 183,0792 found: m/z 183.0793. **The *cis* isomer:** **^1^H NMR** (400 MHz, C_6_D_6_): δ 0.93 (t, *J=*7.1 Hz, 3H), 1.13 (ddd, *J*=23.2 Hz, 6.4 Hz, 2.5 Hz 3H), 2.84 (m,1H), 3.89 (q, *J*=7.1 Hz, 2H), 5.05 (dm, *J*=48.7 Hz, 1H), 5.42-5.52 (m, 1H), 5.60-5.70 (m, 1H); **^13^C NMR** (100 MHz, C_6_D_6_): δ 14.1 (CH_3_), 21.4 (d, *J*=24.9 Hz; CH_3_), 33.1 (CH_2_), 60.5 (CH_2_), 85.4 (d, *J*=161.4 Hz; CH), 125.0 (d, *J*=9.9 Hz, CH), 132.6 (d, *J*=20.3 Hz; CH), 170.5 (C); **^19^F NMR** (376.5 MHz, C_6_D_6_) δ -166.9 (dqdd, *J*=48.7 Hz, 23.2 Hz, 11.8 Hz, 2.5 Hz).

**Ethyl-(*E*)-5-fluoro-2,2-dimethylhex-3-enoate (2c).**

General procedure A. 95% yield, *E/Z >* 20:1. **The *trans* isomer (major):** **^1^H NMR** (400 MHz, C_6_D_6_): δ 0.89 (t, *J*=7.1 Hz, 3H), 1.13 (dd, *J*=23.0 Hz, 6.3 Hz, 3H), 1.23 (s, 6H), 3.89 (q, *J*=7.1 Hz, 2H), 4.79 (dqd, *J*=48.3 Hz, 6.3 Hz, 6.3 Hz, 1H), 5.54 (ddd, *J*=15.9 Hz, 11.8 Hz, 6.3 Hz, 1H); 5.97 (dd., *J*=15.9 Hz, 3.8 Hz, 1H); **^13^C NMR** (100 MHz, C_6_D_6_): δ 14.0 (CH_3_), 21.6 (CH_3_), 21.9 (CH_3_), 25.2 (d, *J*=21.7 Hz; CH_3_), 44.2 (C), 60.9 (CH_2_), 89.8 (d, *J*=164.1 Hz; CH), 128.9 (d, *J*=19.7 Hz; CH), 137.6 (d , *J*=11.1 Hz; CH), 175.5 (C). **^19^F NMR** (376.5 MHz, C_6_D_6_): δ -166.4 (dqdd *J*=48.3 Hz, 23.0Hz, 11.8 Hz, 3.8 Hz); **IR** (neat): 2983, 1732,1471, 1386, 1250, 1145, 1050, 972, 860; **HRMS**: calculated for C_10_H_17_FNaO_2_ m/z 211.1105, found: m/z 211.1105

***syn*-Ethyl 5-fluoro-2-methyl-hex-3-enoate (2e).**

General procedure A. 310mg, 95% yield, *syn/anti* = 19:1, *E/Z (syn)* 15:1, *E/Z (anti)* >20:1. **The trans*-syn*:** **^1^H NMR** (400 MHz, C_6_D_6_): δ 0.90 (t, *J*=7.1 Hz, 3H), 1.09 (dd, *J*=23.3 Hz, 6.3 Hz, 3H), 1.12 (d, *J*=6.8 Hz, 3H), 2.93 (dq, *J*=7.2 Hz, 6.8 Hz, 1H), 3.89 (q, *J*=7.1 Hz, 2H), 4.73 (dqd, *J*=47.8 Hz, 6.3 Hz, 6.3Hz, 1H), 5.46 (ddd., *J*=15.6 Hz, 11.7 Hz, 6.3 Hz, 1H); 5.80 (ddd, *J*=15.6 Hz, 7.2 Hz, 3.9 Hz 1H); **^13^C NMR** (100 MHz, C_6_D_6_): δ 14.1 (CH_3_), 17,1 (CH_3_), 21.4 (d, *J*=24.4 Hz; CH_3_), 42.6 (CH), 60.5 (CH_2_), 89.3 (d, *J*=164.8 Hz; CH), 131.6 (d , *J*=19.7 Hz; CH), 132.0 (d , *J*=11.3 Hz; CH), 173.5 (C). **^19^F NMR** (376.5 MHz, C_6_D_6_): δ -166.6 (dqddd, *J*=47.8 Hz, 23.3Hz, 11.7 Hz, 3.9 Hz, 2.4 Hz); **IR** (neat): 2983, 1737,1457, 1376, 1325, 1260, 1046, 971, 805; **HRMS**: calculated for C_9_H_15_FNaO_2_ m/z 197.0948, found: m/z 197.0946.

***syn-(E*)-Ethyl-2-benzyl-5-fluorohex-3-enoate (2f)**.

General procedure A. 112.0mg, 90% yield, *syn/anti >*20:1, *E/Z (syn)* 11:1, *E/Z (anti)* >20:1. **The *trans-syn* isomer:** **^1^H NMR** (400 MHz, C_6_D_6_): δ 0.93 (t, *J=*7.1 Hz, 3H), 1.15 (dd, *J*=23.2 Hz, 6.6 Hz, 3H), 2.83 (dd, *J*=13.4 Hz, 6.6 Hz, 1H), 3.16 (dd, *J*=13.4 Hz, 6.6 Hz, 1H), 3.37 (dd, *J*=8.1 Hz, 6.6 Hz, 1H), 3.96 (q, *J*=7.1 Hz, 2H), 4.80 (dqd, *J*=48.3 Hz, 6.3 Hz, 6.3 Hz, 1H), 5.53 (ddd., *J*=15.7 Hz, 12.6 Hz, 6.8 Hz, 1H); 5.80 (ddd, *J*=15.7 Hz, 8.6 Hz, 3.5 Hz 1H); **^13^C NMR** (100 MHz, C_6_D_6_): δ 14.0 (CH_3_), 21.2 (d, *J*=24.0 Hz; CH_3_), 39.0 (CH_2_), 50.9 (CH), 60.5 (CH_2_), 88.5 (d, *J*=166.2 Hz; CH), 126.7 (CH), 128.5 (2 x CH), 129.4 (2 x CH), 129.8 (d , *J*=11.2 Hz; CH), 132.0 (d , *J*=19.2 Hz; CH), 172.6 (C). **^19^F NMR** (376.5 MHz, C_6_D_6_): δ -166.6 (dqdd, *J*=48.3 Hz, 23.2 Hz, 12.6 Hz, 3.5 Hz); **IR** (neat): 2982, 1733, 1454, 1260, 1158, 1033, 799, 700; **HRMS**: calculated for C_15_H_19_FNaO_2_ m/z 273.1261, found: m/z 273.1262.

***anti*-(*E)*-Ethyl 5-fluoro-2-methyl-hex-3-enoate (2h).**

General procedure A. 33mg, 96% yield, *syn/anti* 1:6, *E/Z (syn)* >20:1, *E/Z (anti)* 11:1.

**The trans*-anti* isomer: ^1^H NMR** (400 MHz, C_6_D_6_): δ 1.02 (t, *J*=7.3 Hz, 3H), 1.20 (dd, *J*=23.2 Hz, 6.3 Hz, 3H), 1.23 (d, *J*=7.1 Hz, 3H), 3.06 (qd, *J*=7.1 Hz, 2.8 Hz, 1H), 4.01 (q, *J*=7.1 Hz, 2H), 4.85 (dqd, *J*=48.3 Hz, 6.3 Hz, 6.3Hz, 1H), 5.58 (dddd, *J*=15.6 Hz, 11.2 Hz, 6.1, 1.0 Hz, 1H); 5.93 (dddd, *J*=15.7 Hz, 7.6 Hz, 3.8 Hz, 1.0 Hz, 1H); **^13^C NMR** (100 MHz, C_6_D_6_): δ 14.1 (CH_3_), 17,0 (CH_3_), 21.3 (d, *J*=24.8 Hz; CH_3_), 42.5 (CH), 60.5 (CH_2_), 89.2 (d, *J*=164.6 Hz; CH), 131.5 (d , *J*=20.0 Hz; CH), 131.8 (d , *J*=11.2 Hz; CH), 173.5 (C). **^19^F NMR** (376.5 MHz, C_6_D_6_): δ -166.6 (dqddd, *J*=48.1 Hz, 23.1 Hz, 12.0 Hz, 3.8 Hz, 2.7 Hz); **IR** (neat): 2982, 2360, 1736, 1456, 1376, 1259, 1181, 1046, 970, 853, 738; **HRMS**: calculated for C_9_H_15_FNaO_2_ m/z 197.0948, found: m/z 197.0954.

### General Procedure B for electrophilic fluorodesilylation of alcohols.

To a solution of substrate in acetonitrile (0.1 M) was added Selectfluor™ (1.5 eq.) and NaHCO_3_ (1eq.). The reaction was allowed to stir for 4hrs at room temperature. The reaction was diluted with Et_2_O, filtered and the organic phase was washed with water. The organic phase was dried over MgSO_4_, filtered and the solvent removed *in vacuo*. The crude products were purified by rapid silica gel chromatography. (Caution: The corresponding allylic fluoride products are volatile, unstable on silica, alumina (basic and neutral) on prolonged exposure and decompose upon heating).

### (*E*)-5-Fluorohex-3-en-1-ol (2b).

General procedure B. 86mg, 64% yield, *E/Z* = 3:1. **The *trans* isomer:** **^1^H NMR** (400 MHz, C_6_D_6_): δ 1.27 (dd, *J*=23.0 Hz, 6.3 Hz, 3H), 2.03 (br, 1H), 2.14 (td, *J*=6.5 Hz, 6.0Hz, 2H), 3.46 (t, *J*=6.5 Hz, 2H), 4.90 (dqd, *J*=48.6 Hz, 6.3 Hz, 6.3 Hz, 1H), 5.52-5.70 (m, 2H); **^13^C NMR** (100 MHz, C_6_D_6_): δ 21.4 (d, *J*=24.7 Hz, CH_3_), 35.8 (CH_2_), 61.3 (CH_2_), 89.7 (d, *J*=163.2 Hz; CH), 130.0 (d , *J*=11.2 Hz; CH), 132.6 (d , *J*=19.2 Hz; CH); **^19^F NMR** (376.5 MHz, C_6_D_6_): δ -164.6 (m). **HRMS**: calculated for C_6_H_11_FNaO m/z 141.0686, found: m/z 141.0684. **The *cis* isomer:** **^1^H NMR** (400 MHz, C_6_D_6_): δ 1.29 (dd, *J*=23.0 Hz, 6.2 Hz, 3H), 2.03 (br, 1H), 2.14 (td, *J*=6.5 Hz, 6.0 Hz, 2H), 3.41 (t, *J*=6.5 Hz, 2H), 5.32 (dqd, *J*=48.8 Hz, 6.5 Hz, 6.5 Hz, 1H), 5.42-5.52 (m, 1H); 5.52-5.70 (m, 1H); **^13^C NMR** (100 MHz, C_6_D_6_): δ 21.7 (d, *J*=25.2 Hz; CH_3_), 31.4 (CH_2_), 65.9 (CH_2_), 85.4 (d, *J*=159.9 Hz; CH), 130.1 (d , *J*=10.4 Hz; CH), 132.1 (d , *J*=20.4 Hz; CH): **^19^F NMR** (376.5 MHz, C_6_D_6_): δ -166.5 (m).

**(*E*)-5-Fluoro-2,2-dimethylhex-3-enoate (2d).**

General procedure B. Purification by silica gel chromatography (20-50% Et_2_O/hexane) 46mg, 70% yield, *E/Z* > 20:1. **The *trans-* isomer:** **^1^H NMR** (400 MHz, C_6_D_6_): δ 0.87 (s, 3H), 0.88 (s, 3H), 1.18 (dd, *J*=23.0 Hz, 6.3 Hz, 3H), 1.94 (br, 1H), 3.13 (s, 2H), 4.83 (dqdd, *J*=48.4 Hz, 6.3 Hz, 6.3 Hz, 0.7 Hz, 1H), 5.47 (ddd, *J*=15.9 Hz, 10.6 Hz, 6.3 Hz, 1H), 5.61 (ddd, *J*=15.9 Hz, 3.9 Hz, 0.7 Hz, 1H); **^13^C NMR** (100 MHz, C_6_D_6_): δ 21.3 (d, *J*=24.8 Hz; CH_3_), 23.3 (CH_3_), 23.4 (CH_3_), 37.8 (C), 71.0 (d, *J*=2.3 Hz, CH_2_), 90.3 (d, *J*=163.6 Hz; CH), 128.2 (d , *J*=19.4 Hz, CH), 140.0 (d , *J*=10.7 Hz, CH); **^19^F NMR** (376.5 MHz, C_6_D_6_): δ -164.7 (dqdd, *J*=48.4 Hz, 23.0 Hz, 10.6 Hz, 3.9 Hz); **IR** (neat): 3386, 2963, 1379, 1048, 975, 856; **HRMS**: calculated for C_8_H_15_FNaO m/z 169.0999, found: m/z 169.1001.

***syn*-(*E*)-5-Fluoro-2-methylhex-3-en-1-ol (2g).**

General procedure B. Purification by silica gel chromatography (20-50% Et_2_O/hexane) 87mg, 66% yield, *syn/anti* >20:1, *E/Z* (*syn*) 15:1, *E/Z* (*anti*) >20:1. **The *trans-syn* isomer:** **^1^H NMR** (400 MHz, C_6_D_6_): δ 0.86 (d, *J*=6.8 Hz, 3H), 1.18 (dd, *J*=23.0 Hz, 6.3 Hz, 3H), 1.96 (br, 1H), 2.08-2.18 (m, 1H), 3.23 (t, *J*=6.5 Hz, 2H), 4.81 (dqd, *J*=47.6 Hz, 6.3 Hz, 5.9 Hz, 1H), 5.43-5.56 (m, 2H); **^13^C NMR** (100 MHz, C_6_D_6_): δ 16.6 (CH_3_), 22.0 (d, *J*=24.7 Hz, CH_3_), 39.6 (CH), 67.5 (CH_2_), 90.2 (d, *J*=163.5 Hz, CH), 131.0 (d , *J*=19.3 Hz, CH), 136.2 (d , *J*=10.8 Hz; CH); **^19^F NMR** (376.5 MHz, C_6_D_6_): δ -164.7 (dqdd, *J*=47.6 Hz, 23.0 Hz, 8.2 Hz, 4.3 Hz); **IR** (neat): 3374, 2930, 1377, 1041, 972, 844; **HRMS**: calculated for C_7_H_13_FNaO m/z 155.0843, found: m/z 155.0842.

***anti*-(*E*)-5-Fluoro-2-methylhex-3-en-1-ol (2i).**

General procedure B. 11.5mg, 86% yield, *syn/anti* 1:8, *E/Z* (*syn*) 14:1, *E/Z* (*anti*) 10:1. **The *trans-anti* isomer: ^1^H NMR** (400 MHz, C_6_D_6_): δ 0.92 (d, *J*=6.8 Hz, 3H), 1.27 (dd, *J*=23.0 Hz, 6.3 Hz, 3H), 2.16-2.22 (m, 1H), 3.29 (d, *J*=6.6 Hz, 2H), 4.89 (dm, *J*=48.3 Hz, 1H), 5.43-5.56 (m, 2H); **^13^C NMR** (100 MHz, C_6_D_6_): δ 16.2 (CH_3_), 21.5 (d, *J*=24.8 Hz, CH_3_), 39.2 (CH), 67.0 (CH_2_), 89.7 (d, *J*=163.8 Hz, CH), 130.7 (d , *J*=19.2 Hz, CH), 135.8 (d , *J*=10.4 Hz; CH); **^19^F NMR** (376.5 MHz, C_6_D_6_): δ -164.7 (dqdd, *J*=51.7 Hz, 23.0 Hz, 7.1 Hz, 2.8 Hz); **IR** (neat): 3385, 2962, 2927, 2281, 1455, 1377, 1037, 802; **HRMS**: calculated for C_7_H_13_FNaO m/z 155.0843, found: m/z 155.0844.

**(*E*)-Ethyl 6-(benzyloxy)-3-(trimethylsilyl)hex-4-enoate (4).**

A mixture of (*E*)-1-(benzyloxy)-4-(trimethylsilyl)but-3-en-2-ol (735mg, 2.95mmol, 1eq.), triethylacetate (1.08ml, 5.9mmol, 2eq.) and catalytic propionic acid in toluene was allowed to reflux for 3 days. After that time the mixture was cooled to room temperature. The crude reaction mixture was diluted with hexane (15ml) and purified directly by silica gel chromatography (100% hexane to hexane:Et_2_O 2:1) to afford (**4**) 700 mg, 74% yield. **^1^H NMR** (400 MHz, acetone-d_6_): δ 0.05 (s, 9H), 1.20 (t, *J*=7.1 Hz, 3H), 2.10 (td, *J*=9.4 Hz, 4.8 Hz, 3H), 2.38 (dd, *J*=15.2 Hz, 10.4 Hz, 1H), 2.46 (dd, *J*=15.2 Hz, 4.8 Hz, 1H), 3.97 (d, *J*=6.1 Hz, 2H), 4.07 (q, *J*=7.1 Hz, 2H), 4.46 (s, 2H), 5.45 (dt, *J*=15.4 Hz, 6.3 Hz, 1H), 5.69 (dd, *J*=15.4 Hz, 8.8 Hz, 1H), 7.24-7.30 (m, 1H), 7.33-7.35 (m, 4H); **^13^C NMR** (100 MHz, acetone-d_6_): δ -3.6 (3 x CH_3_), 14.2 (CH_3_), 29.7 (CH), 34.1 (CH_2_), 60.1 (CH_2_), 70.9 (CH_2_), 71.1 (CH_2_), 125.0 (CH), 127.6 (CH), 127.8 (2 x CH), 128.5 (2 x CH), 134.7 (CH), 139.6 (C), 172.9 (C); **IR** (neat): 2955, 1736, 1249, 1097, 841, 737; **HRMS**: calculated for C_18_H_28_NaO_3_Si m/z 343.1700, found: m/z 343.1701.

**(*E*)-ethyl 6-(benzyloxy)-2-fluoro-3-(trimethylsilyl)hex-4-enoate (6).**

To a solution of diisopropylamine (430uL, 2.6mmol, 1.3eq.) in THF (2 ml) at 0°C was added *n*-BuLi (2.5M, 1.2ml, 2.4mmol, 1.2eq.) dropwise. The mixture was allowed to stir at this temperature for 30 min. The reaction was then cooled to –78°C and (*E*)-ethyl-6-(benzyloxy)-3-(trimethylsilyl)hex-4-enoate (**4**) (640mg, 2.0mmol, 1eq.) in THF (2 mL) was added dropwise. The reaction was allowed to stir at –78°C for 1hr. After this time *N*-fluorobenzenesulfonimide (NFSI) (950mg, 3.0mmol, 1.5eq.) in THF (2 mL) was added dropwise and the mixture allowed to warm room temperature overnight. The reaction was then cooled to 0°C and quenched by the addition of a saturated solution of NH_4_Cl. The organic phase was separated and aqueous phase extracted with Et_2_O. The combined organic extracts were dried over MgSO_4_, filtered and the solvent removed *in vacuo*. Purification by silica gel chromatography gave (**6**) 380mg 56% yield, d.r >20:1. **^1^H NMR** (400 MHz, acetone-d_6_): δ 0.14 (s, 9H), 1.23 (t, *J*=7.1 Hz, 3H), 2.28 (ddd, *J*=45.0 Hz, 10.4 Hz, 3.0 Hz, 1H), 3.97 (dd, *J*=12.4 Hz, 6.1 Hz, 1H), 4.01 (dd, *J*=11.9 Hz, 5.3 Hz, 1H), 4.15 (dq, *J*=10.9 Hz, 7.1 Hz, 1H), 4.21 (dq, *J*=10.9 Hz, 7.1 Hz, 1H), 4.48 (s, 2H), 5.21 (dd, *J*=47.0 Hz, 3.0 Hz, 1H), 5.29 (dd, *J*=15.4 Hz, 6.0 Hz, 1H), 7.24-7.31 (m, 1H), 7.33-7.37 (m, 4H); **^13^C NMR** (100 MHz, acetone-d_6_): δ -2.8 (3 x CH_3_), 14.3 (CH_3_), 37.8 (d, *J*=25.6 Hz, CH), 61.1 (CH_2_), 70.6 (CH_2_), 71.3 (CH_2_), 90.2 (d, *J*=182.2 Hz, CH), 127.7 (CH), 127.8 (2 x CH), 128.4 (CH), 128.6 (2 x CH), 128.8 (CH), 139.4 (C), 169.4 (d, *J*=27.2 Hz, C); **^19^F NMR** (376.5 MHz, acetone-d_6_): δ –196.1 (dtd, *J*=47.3 Hz, 26.9 Hz, 9.9 Hz, 1F), -191.3 (pt, *J*=46.1 Hz, 1F), **IR** (neat): 2957, 1760, 1250, 1205, 1097, 843, 738; **HRMS**: calculated for C_18_H_27_F_1_NaO_3_Si_1_ m/z 361.1606, found: m/z 361.1605.

**(*E*)-Ethyl 6-(benzyloxy)-2,5-difluorohex-3-enoate (3).**

To a solution of (*E*)-ethyl-6-(benzyloxy)-2-fluoro-3-(trimethylsilyl)hex-4-enoate (**6**) (340mg, 1.0mmol, 1eq) in acetonitrile (10ml) was added Selectfluor™ (700mg, 2.5mmol, 2.5 eq.) and NaHCO_3_ (84mg, 1.0mmol, 1eq.) at 50°C. The reaction was allowed to stir at this temperature for 12hrs. The reaction mixture was cooled to room temperature, diluted with Et_2_O, filtered and the organic phase washed with water. The organic phase was dried over MgSO_4_, filtered and the solvent removed *in vacuo* to give (**3**) as colourless oil, 280mg 98% yield, *syn/anti* = 5:1, *E/Z* (*syn*) 6:1, *E/Z* (*anti*) 1:1. **^1^H NMR** (400 MHz, acetone-d_6_): δ 1.26 (t, *J*=7.1 Hz, 3H), 3.66 (ddd, *J*=22.5 Hz, 11.4 Hz, 6.1 Hz, 1H), 3.71 (ddd, *J*=26.0 Hz, 11.4 Hz, 3.3Hz, 1H), 4.23 (q, *J*=7.1 Hz, 2H), 4.60 (s, 2H), 5.26 (dm, *J*=49.0 Hz, 1H), 5.53 (dm, *J*=47.8, 1H), 6.02-6.22, (m, 2H), 7.27-7.32 (m, 1H), 7.34-7.39 (m, 4H); **^13^C NMR** (100 MHz, acetone-d_6_): δ 13.9 (CH_3_), 61.8 (CH_2_), 72.0 (dd, *J*=22.4 Hz, 1.6 Hz, CH_2_), 73.3 (CH_2_), 87.8 (d, *J*=182.1 Hz, CH), 91.3 (d, *J*=172.6 Hz, CH), 128.0 (CH), 128.1 (2 x CH), 128.7 (2 x CH), 131.0 (dd, J=19.2 Hz, 11.2Hz, CH), 138.8 (C), 167.9 (dd, *J*=24.8 Hz, 1.6 Hz, C); **^19^F NMR** (376.5 MHz, acetone-d_6_): δ –188.4 (ddm, *J*=47.4 Hz, 14.7 Hz, 1F), -186.3 (m, 1F), **IR** (neat): 2937, 1761, 1453, 1371, 1274, 1207, 1115, 1027, 970, 740. 700; **HRMS**: calculated for C_15_H_18_FNaO_3_ m/z 307.1116, found: m/z 307.1110.

**Ethyl 6-(benzyloxy)-2,5-(*syn*)-difluoro-3,4-(*anti*)-dihydroxyhexanoate (8).**

To a solution of *N*-methylmorpholine (246mg, 2.1mmol, 3eq), osmium tetraoxide (9mg, 0.05eq) in water (38uL) and DCM (3.5 mL) was added (*E*)-ethyl-6-(benzyloxy)-2,5-difluorohex-3-enoate (**3**) (200mg, 0.7mmol, 1eq) in DCM (3.5 mL). The mixture was allowed to stir at room temperature for 3hrs. A saturated solution of NaHSO_3_ was then added and stirred for a further 15 min. DCM was added and the phases separated. The aqueous phase was extracted with EtOAc and the combined organic phases were dried over Na_2_SO_4_, filtered and the solvent removed *in vacuo* Purification by silica gel chromatography (EtOAc:hexane 2:1, R_f_ = 0.5) to yield (**8**) 126mg, 56% yield. **NMR data of the major diastereoisomer: ^1^H NMR** (400 MHz, acetone-d_6_): δ 1.47 (t, *J*=7.1 Hz, 3H), 4.03 (ddd, *J*=30.1 Hz, 11.9 Hz, 5.1 Hz, 1H), 4.13 (ddd, *J*=25.0 Hz, 11.9 Hz, 2.0 Hz, 1H), 4.18-4.24 (m, 1H), 4.26-4.33 (m, 1H), 4.44 (q, *J*=7.1 Hz, 2H), 4.65 (d, *J*=7.8, 1H), 4.72, (d, *J*=8.1 Hz, 1H), 4.79 9s, 2H), 4.92 (dm, *J*=47.5 Hz, 1H), 5.14 (dd, *J*=48.5 Hz, 8.1 Hz, 1H), 7.46-7.50 (m, 1H), 7.53-7.58 (m, 4H); **^13^C NMR** (100 MHz, acetone-d_6_): δ 14.1 (CH_3_), 61.7 (CH_2_), 67.5 (d, *J*=24.8 Hz, CH_2_), 69.4 (dd, J=24.8 Hz, 3.2 Hz, CH), 70.1 (d, *J*=19.2 Hz, CH), 73.5 (CH_2_), 88.7 (d, *J*=182.2 Hz, CH), 91.5 (d, *J*=175.0 Hz, CH), 128.0 (CH), 128.1 (2 x CH), 128.8 (2 x CH), 139.2 (C), 168.9 (d, *J*=22.4 Hz, C); **^19^F NMR** (376.5 MHz, acetone-d_6_): δ –194.1 (ddddd, *J*=47.6 Hz, 29.6 Hz, 27.1 Hz, 5.1 Hz, 2.5 Hz, 1F), -196.1 (ddd, *J*=48.5 Hz, 9.7 Hz, 2.3 Hz 1F), **HRMS**: calculated for C_15_H_20_F_2_NaO_5_ m/z 341.1171, found: m/z 341.1170.

### Ethyl-6-(benzyloxy)-2,5-(*syn*)-difluoro-3-O-,4-O-(*anti*)-isopropylidene-hexanoate (9).

To a solution of ethyl-6-(benzyloxy)-2,5-(*syn*)-difluoro-3,4-(*anti*)-dihydroxyhexanoate (**8**) (95mg, 0.3mmol, 1eq.) in acetone (1.5ml) was added dimethoxypropane (1.5ml) and *p*-TSA (6mg). The mixture was allowed to stir at room temperature overnight. The solvent was removed *in vacuo* and the crude product purified by silica gel chromatography (1:1 Et_2_O:hexane, R_f_ = 0.6) to yield (**9**) 96mg, 90% yield as a pale yellow oil. **NMR data of the major diastereoisomer: ^1^H NMR** (400 MHz, acetone-d_6_): δ 1.28 (t, *J*=7.3 Hz, 3H), 1.37 (s, 3H), 1.38 (s, 3H), 3.75 (ddd, *J*=28.3 Hz, 11.9 Hz, 5.1 Hz, 1H), 3.85 (ddd, *J*=26.3 Hz, 11.9 Hz, 2.5 Hz, 1H), 4.23 (q, *J*=7.1 Hz, 2H), 4.24 (q, *J*=7.1 Hz, 2H), 4.46 (dt, *J*=9.9 Hz, 6.8 Hz, 1H), 4.53-4.63 (m, 1H), 4.76 (dddd, J=47.5 Hz, 6.8 Hz, 5.3 Hz, 2.5 Hz, 1H), 5.24 (dd, *J*=48.0 Hz, 2.3 Hz, 1H), 7.28-7.32 (m, 1H), 7.35-7.40 (m, 4H); **^13^C NMR** (100 MHz, acetone-d_6_): δ 13.9 (CH_3_), 26.6 (CH_3_), 26.9 (CH_3_), 61.7 (CH_2_), 69.7 (d, *J*=20.0 Hz, CH_2_), 73.4 (CH_2_), 74.4 (dd, *J*=27.2 Hz, 7.2 Hz, CH), 79.5 (dd, *J*=22.4 Hz, 3.2 Hz, CH), 88.6 (d, *J*=189.3 Hz, CH), 93.1 (d, *J*=175.0 Hz, CH), 111.3 (C), 127.9 (CH), 128.0 (2 x CH), 128.7 (2 x CH), 138.7 (C), 166.8 (d, *J*=24.0 Hz, C); **^19^F NMR** (376.5 MHz, acetone-d_6_): δ –196.1 (dtd, *J*=47.3 Hz, 26.9 Hz, 9.9 Hz, 1F), -198.4 (dd, J=48.0 Hz, 22.1 Hz, 1F), **HRMS**: calculated for C_18_H_24_F_2_NaO_5_ m/z 381.1484, found: m/z 381.1484.

**Ethyl 6-(benzyloxy)-2,5-(*syn*)-difluoro-3-O-,4-O-*anti*-isopropylidene-1-hexanol (10).**

To suspension of LiAlH_4_ (38mg, 1mmol) in Et_2_O (2.5 mL) at 0°C was added ethyl-6-(benzyloxy)-2,5-(*syn*)-difluoro-3-O-,4-O-(*anti*)-isopropylidene-hexonate (**9**) (90mg, 0.25mmol, 1eq) in Et_2_O (2.5 mL) dropwise. The reaction was then allowed to stir at 0°C for 3hrs. The reaction was quenched by the addition of a saturated solution of NH_4_Cl, and the aqueous phase extracted with Et_2_O. The combined organic extracts dried over Na_2_SO_4_, filtered and the solvent removed *in vacuo*. Purification by silica gel chromatography (1:1 Et_2_O:hexane R_f_ = 0.3), to give (**10**) 67mg 85 % yield as a mixture of stereoisomers. **NMR data of the major diastereoisomer: ^1^H NMR** (400 MHz, acetone-d_6_): δ 1.38 (s, 3H), 1.39 (s, 3H), 3.72-3.92 (m, 4H), 4.12 (t, *J*=6.0 Hz, 1H), 4.34 (dt, *J*=11.7 Hz, 6.0 Hz, 1H), 4.38 (dt, *J*=12.3 Hz, 6.0 Hz, 1H), 4.59 (dtd, *J*=48.2 Hz, 5.7 Hz, 2.8 Hz, 1H), 4.61 (s, 2H), 4.80 (dtd, *J*=48.2 Hz, 5.7 Hz, 2.8 Hz), 7.29-7.32 (m, 1H), 7.35-7.40 (m, 4H); **^13^C NMR** (100 MHz, acetone-d_6_): δ 27.5 (2 x CH_3_), 60.9 (d, *J*=22.9 Hz, CH_2_), 69.9 (d, *J*=21.0 Hz, CH_2_), 73.8 (CH_2_), 77.1 (dd, *J*=25.8 Hz, 4.8 Hz, CH), 77.6 (dd, *J*=24.8 Hz, 3.8 Hz, CH), 93.3 (d, *J*=176.4 Hz, CH), 95.0 (d, *J*=174.5 Hz, CH), 111.3 (C), 128.4 (CH), 128.5 (2 x CH), 129.2 (2 x CH), 139.3 (C); **^19^F NMR** (376.5 MHz, acetone-d_6_): δ –194.7 (m, 1F), -197.4 (m, 1F); **IR** (neat): 3442, 2938, 1455, 1373, 1217, 1069, 867; **HRMS**: calculated for C_16_H_22_F_2_NaO_4_ m/z 339.1378, found: m/z 339.1384.

**4,5-Bis(2-(benzyloxy)-1-fluoroethyl)-2,2-dimethyl-1,3-dioxolane (11).**

To a suspension of NaH (23mg, 0.6mmol, 3eq) in DMF (1 ml) at 0°C was added ethyl-6-(benzyloxy)-2,5-(*syn*)-difluoro-3-O-,4-O-(*anti*)-isopropylidene-1-hexanol (**10**) (63mg, 0.2mmol, 1eq) DMF (1 mL). The mixture was allowed to stir at 0°C for 1hr after which time benzyl bromide (75uL, 0.6mmol, 3eq) was added dropwise. The reaction mixture was then heated at 40°C for 6hr. After cooling to 0°C the reaction was quenched by the addition of water. The aqueous phase was extracted with DCM, the combined organic extracts were dried over Na_2_SO_4_, filtered and the solvent removed *in vacuo*. Purification by silica gel chromatography (1:1 DCM:hexane) to yield (**11**) 63mg 78% yield as a mixture of stereoisomers. **NMR data of the major diastereoisomer: ^1^H NMR** (400 MHz, acetone-d_6_): δ 1.38 (s, 6H), 3.75 (ddd, *J*=24.6 Hz, 11.7 Hz, 6.0 Hz, 2H), 3.83 (ddd, *J*=27.1 Hz, 11.7 Hz, 2.5 Hz, 2H), 4.12-4.40 (m, 2H), 4.61 (s, 4H), 4.80 (dm, *J*=48.2 Hz, 2H), 7.29-7.32 (m, 2H), 7.35-7.40 (m, 8H); **^13^C NMR** (100 MHz, acetone-d_6_): δ 27.5 (2 x CH_3_), 69.9 (d, *J*=22.9 Hz, 2 x CH_2_), 73.9 (2 x CH_2_), 77.4 (dd, *J*=25.8 Hz, 2.9 Hz, 2 x CH), 93.3 (d, *J*=175.5 Hz, 2 x CH), 111.5 (2 x C), 128.4 (2 x CH), 128.5 (4 x CH), 129.2 (4 x CH), 139.3 (2 x C); **^19^F NMR** (376.5 MHz, acetone-d_6_): δ –194.5 (dtd, *J*=47.7 Hz, 26.8 Hz, 2.6 Hz, 1F), **IR** (neat): 2938, 1453, 1371, 1068, 736; **HRMS**: calculated for [M+NH_4_] C_23_H_32_F_2_N_1_O_4_ m/z 424.2292, found: m/z 424.2294.

**1,6-bis(benzyloxy)-2,5-difluorohexane-3,4-diol (7).**

A solution of 4,5-bis(2-(benzyloxy)-1-fluoroethyl)-2,2-dimethyl-1,3-dioxolane (**11**) (41mg, 0.1mmol) in AcOH:H_2_O 7:3 (1 mL) and was heated at 100°C for 24hrs. The reaction was allowed to cool and toluene was added. The solvents were removed *in vacuo*. Purification by silica gel chromatography (1:1 EtOAc:hexane) to give (**7**) 30 mg, 82% yield as a mixture of stereoisomers. **NMR data of the major diastereoisomer:** **^1^H NMR** (400 MHz, CDCl_3_): δ 2.77 (d, *J*=7.3, 2H), 3.81 (ddd, *J*=27.4 Hz, 11.4 Hz, 4.4 Hz, 2H), 3.88 (ddd, *J*=27.1 Hz, 11.4 Hz, 3.5 Hz, 2H), 4.08 (dd, *J*=7.9 Hz, 7.3 Hz, 2H), 4.59 (d, *J*=11.9 Hz, 2H), 4.64 (d, *J*=11.9 Hz, 2H),4.67 (dm, *J*=46.5 Hz, 2H), 7.30-7.38 (m, 10H); **^13^C NMR** (100 MHz, CDCl_3_): δ 68.6 (dd, *J*=24.8 Hz, 3.8 Hz, 2 x CH), 69.5 (d, *J*=21.9 Hz, 2 x CH_2_), 74.1 (2 x CH_2_), 90.7 (d, *J*=174.5 Hz, 2 x CH), 128.1 (4 x CH), 128.3 (2 x CH), 128.8 (4 x CH), 137.7 (2 x C); **^19^F NMR** (376.5 MHz, CDCl_3_): δ –194.8 (dddd, *J*=47.7 Hz, 28.3 Hz, 21.4 Hz, 7.9, 2F), The major stereoisomer was identical in all aspects with sample prepared by previously published method.^2^

References:

1. a) G. Procter, A. T. Russel, P. J. Murphy, T. S. Tan, A. N. Mather, *Tetrahedron*, **1988**, *44*, 3953; b) M. A. Sparks, J. S. Panek, *J. Org. Chem.* **1991**, *56*, 3431; c) J. S. Panek, R. Beresis, F. Xu, M. Yang, *J. Org. Chem.* **1991**, *56*, 7341; d) J. S. Panek, M. Yang, J. S. Solomon, *J. Org. Chem.* **1993**, *58*, 1003; e) C. E. Masse, B. S. Knight, P. Stavropoulos, J. S. Panek, *J. Am. Chem. Soc.* **1997**, *119*, 6040.

2. L. Hunter, D. O’Hagan, A. M. Z. Slawin, *J. Am. Chem. Soc.* **2006**, *128*, 16422.
